# Supplementary material for: Mechanisms governing the pioneering and redistribution capabilities of the non-classical pioneer PU.1
Source: Nat Commun. 2020 Jan 21;11:402. doi: 10.1038/s41467-019-13960-2 (PMC6972792; doi:10.1038/s41467-019-13960-2)
Supplement: Supplementary file 7 — Source data [file 41467_2019_13960_MOESM7_ESM.zip › Source_Data/FigureS2/FigureS2B_MotifScanOutput/KmeansCluster14/homerResults.html]

/misc/data/analysis/project\_PU1/CTV1/motifs/basic/PU1\_Kmeans14.14/final - Homer de novo Motif Results


# Homer *de novo* Motif Results (/misc/data/analysis/project\_PU1/CTV1/motifs/basic/PU1\_Kmeans14.14/final)

Known Motif Enrichment Results  
Gene Ontology Enrichment Results  
If Homer is having trouble matching a motif to a known motif, try copy/pasting the matrix file into
STAMP  
More information on motif finding results: HOMER
| Description of Results
| Tips
  
Total target sequences = 510  
Total background sequences = 45297  
\* - possible false positive  

|  |  |  |  |  |  |  |  |  |
| --- | --- | --- | --- | --- | --- | --- | --- | --- |
| Rank | Motif | P-value | log P-pvalue | % of Targets | % of Background | STD(Bg STD) | Best Match/Details | Motif File |
| 1 | C T G A T C A G T G A C C T A G A C T G C G T A C G T A T C A G A G C T A T C G T A C G C T G A | 1e-153 | -3.538e+02 | 64.90% | 13.56% | 41.5bp (69.9bp) | ETV6/MA0645.1/Jaspar(0.956) More Information \| Similar Motifs Found | motif file (matrix) |
| 2 | C T G A T C A G A G T C A G T C C G T A G T C A G A C T T A G C T C G A T C A G T G C A C T A G | 1e-65 | -1.517e+02 | 26.08% | 3.95% | 56.4bp (65.5bp) | NFY(CCAAT)/Promoter/Homer(0.963) More Information \| Similar Motifs Found | motif file (matrix) |
| 3 | G C A T C G T A C G T A A T G C G T A C C T G A A G T C C G T A | 1e-48 | -1.126e+02 | 28.82% | 6.96% | 53.7bp (64.6bp) | RUNX(Runt)/HPC7-Runx1-ChIP-Seq(GSE22178)/Homer(0.973) More Information \| Similar Motifs Found | motif file (matrix) |
| 4 | T C A G T G A C A T C G G T A C A T C G C T A G G C T A G C T A | 1e-34 | -7.911e+01 | 29.61% | 9.85% | 52.8bp (79.0bp) | Elk1(ETS)/Hela-Elk1-ChIP-Seq(GSE31477)/Homer(0.863) More Information \| Similar Motifs Found | motif file (matrix) |
| 5 | G T C A A G T C A G T C T C G A A T G C T C G A C T G A C G A T C A G T A G T C A G T C A G T C | 1e-27 | -6.367e+01 | 10.78% | 1.53% | 51.1bp (68.7bp) | GFY(?)/Promoter/Homer(0.917) More Information \| Similar Motifs Found | motif file (matrix) |
| 6 | A G C T C G A T A C G T A G T C T G A C A C G T A T C G A C G T | 1e-27 | -6.327e+01 | 22.16% | 6.84% | 51.2bp (67.1bp) | EWS:FLI1-fusion(ETS)/SK\_N\_MC-EWS:FLI1-ChIP-Seq(SRA014231)/Homer(0.926) More Information \| Similar Motifs Found | motif file (matrix) |
| 7 | A C T G C T A G G T A C T C A G C A T G C A T G T G C A G T A C C G A T C G T A G T C A | 1e-22 | -5.074e+01 | 16.86% | 4.93% | 51.9bp (70.2bp) | UGA3/MA0410.1/Jaspar(0.815) More Information \| Similar Motifs Found | motif file (matrix) |
| 8 | G A T C T G A C A T C G T G A C A G T C G A C T G T C A G A C T | 1e-21 | -4.934e+01 | 38.24% | 19.62% | 58.7bp (70.2bp) | PB0164.1\_Smad3\_2/Jaspar(0.738) More Information \| Similar Motifs Found | motif file (matrix) |
| 9 | T C G A T G C A T G C A C G A T T A C G C G T A A C T G A T C G C G T A C T G A T C A G T A C G A T G C | 1e-20 | -4.612e+01 | 9.02% | 1.55% | 37.0bp (67.3bp) | SPIC/MA0687.1/Jaspar(0.775) More Information \| Similar Motifs Found | motif file (matrix) |
| 10 | C A G T C T A G A G T C A T C G C A T G A G C T A G C T C G A T T G C A | 1e-19 | -4.544e+01 | 13.33% | 3.49% | 60.6bp (73.3bp) | RUNX2/MA0511.2/Jaspar(0.893) More Information \| Similar Motifs Found | motif file (matrix) |
| 11 | A C T G A G T C C A G T A C T G G A T C A G C T T G C A | 1e-19 | -4.430e+01 | 32.75% | 16.20% | 57.1bp (69.9bp) | UME6/UME6\_YPD/51-UME6(Harbison)/Yeast(0.765) More Information \| Similar Motifs Found | motif file (matrix) |
| 12 | T A G C C G T A T A C G C G T A A C G T G T C A C G T A A C T G T G C A | 1e-18 | -4.338e+01 | 9.22% | 1.74% | 52.1bp (63.8bp) | Gata6(Zf)/HUG1N-GATA6-ChIP-Seq(GSE51936)/Homer(0.952) More Information \| Similar Motifs Found | motif file (matrix) |
| 13 | A T C G T A C G G T C A A G C T A C T G A G C T A C T G A G T C T C A G A C T G C A G T A G C T C A G T A G T C | 1e-18 | -4.217e+01 | 10.20% | 2.22% | 49.9bp (65.9bp) | RUNX3/MA0684.1/Jaspar(0.770) More Information \| Similar Motifs Found | motif file (matrix) |
| 14 | A G C T G A C T A G T C T A G C C A T G A G T C A T G C G A T C A G T C A G C T G A C T A G C T A G T C | 1e-17 | -4.079e+01 | 5.69% | 0.61% | 52.3bp (78.1bp) | MSN2/MSN2\_H2O2Hi/1-MSN2(Harbison)/Yeast(0.855) More Information \| Similar Motifs Found | motif file (matrix) |
| 15 | A T G C G T C A T A G C A G C T C G A T A G T C G T A C A T C G A T G C A C G T A C G T A G T C G A T C A C T G | 1e-17 | -4.025e+01 | 5.10% | 0.47% | 56.4bp (65.6bp) | SpiB(ETS)/OCILY3-SPIB-ChIP-Seq(GSE56857)/Homer(0.767) More Information \| Similar Motifs Found | motif file (matrix) |
| 16 | T C A G A G T C A G T C C T G A T A G C G T C A G T C A T A G C C T A G T G C A G T A C A G T C C G T A G T A C | 1e-17 | -3.975e+01 | 10.78% | 2.63% | 48.1bp (61.1bp) | TCP16(TCP)/colamp-TCP16-DAP-Seq(GSE60143)/Homer(0.619) More Information \| Similar Motifs Found | motif file (matrix) |
| 17 | A G T C A C G T A G T C A G C T A C G T G T A C A G T C A G T C G A C T A G C T G A T C G C T A C T G A T A C G | 1e-16 | -3.848e+01 | 10.78% | 2.71% | 50.3bp (69.7bp) | SRSF1(RRM)/Homo\_sapiens-RNCMPT00163-PBM/HughesRNA(0.708) More Information \| Similar Motifs Found | motif file (matrix) |
| 18 | A C G T C T A G G A C T C T A G C T A G A G T C G C T A A C T G G A C T | 1e-16 | -3.831e+01 | 20.78% | 8.55% | 58.0bp (63.5bp) | RUNX2(Runt)/PCa-RUNX2-ChIP-Seq(GSE33889)/Homer(0.725) More Information \| Similar Motifs Found | motif file (matrix) |
| 19 | G C A T A C T G C G T A A G T C A C G T A T G C C G T A | 1e-16 | -3.766e+01 | 27.25% | 13.17% | 57.2bp (69.6bp) | GCN4/GCN4\_SM/121-GCN4(Harbison)/Yeast(0.953) More Information \| Similar Motifs Found | motif file (matrix) |
| 20 | T A G C T A G C C A T G C A G T G C T A G C T A T A G C | 1e-16 | -3.722e+01 | 42.94% | 25.84% | 55.7bp (73.7bp) | MYB98(MYB)/col-MYB98-DAP-Seq(GSE60143)/Homer(0.826) More Information \| Similar Motifs Found | motif file (matrix) |
| 21 | A C T G A G C T C G T A C G T A A G T C A G T C C T A G G T C A | 1e-15 | -3.556e+01 | 10.39% | 2.71% | 49.9bp (71.9bp) | GAMYB(MYB)/Hordeum vulgare/AthaMap(0.843) More Information \| Similar Motifs Found | motif file (matrix) |
| 22 | C A G T C A T G G C A T A C T G A C G T C A G T T A G C G A C T C T A G A T C G A C G T | 1e-14 | -3.439e+01 | 3.14% | 0.15% | 57.1bp (69.0bp) | RBM38(RRM)/Mus\_musculus-RNCMPT00051-PBM/HughesRNA(0.715) More Information \| Similar Motifs Found | motif file (matrix) |
| 23 | C G A T C G T A A C T G G T A C C G T A C G T A G T A C T G A C C A T G A G T C | 1e-14 | -3.271e+01 | 3.73% | 0.29% | 48.0bp (66.0bp) | RFX1/MA0365.1/Jaspar(0.814) More Information \| Similar Motifs Found | motif file (matrix) |
| 24 | A C T G A C T G C A T G A T G C A C G T A C G T A T G C G A T C A G C T A T G C A G C T G C A T A G T C T G A C | 1e-14 | -3.239e+01 | 5.88% | 0.93% | 49.3bp (69.8bp) | GCR1/Literature(Harbison)/Yeast(0.788) More Information \| Similar Motifs Found | motif file (matrix) |
| 25 | C T G A C T G A C T A G T C A G T A C G A T C G T A C G T A C G C T A G | 1e-13 | -3.208e+01 | 28.82% | 15.33% | 57.2bp (68.2bp) | MSN2/MSN2\_H2O2Hi/1-MSN2(Harbison)/Yeast(0.895) More Information \| Similar Motifs Found | motif file (matrix) |
| 26 | A C T G A C T G A C T G G T A C A C T G A C T G C T A G C T A G A G C T A G T C | 1e-13 | -3.203e+01 | 15.10% | 5.66% | 56.5bp (66.5bp) | POL003.1\_GC-box/Jaspar(0.908) More Information \| Similar Motifs Found | motif file (matrix) |
| 27 | A C T G A G C T A C T G C A G T C A T G C T A G A G C T C G A T A G C T A G T C | 1e-13 | -3.012e+01 | 10.20% | 3.02% | 58.8bp (70.8bp) | che-1/MA0260.1/Jaspar(0.821) More Information \| Similar Motifs Found | motif file (matrix) |
| 28 | C G A T A C G T G C T A A C G T A G T C G C T A A T C G C G T A A G C T G T C A C G T A C T A G | 1e-13 | -3.000e+01 | 2.75% | 0.13% | 52.8bp (67.3bp) | PB0022.1\_Gata5\_1/Jaspar(0.733) More Information \| Similar Motifs Found | motif file (matrix) |
| 29 | A C G T A C G T A G T C A G T C A G T C A G T C A G T C | 1e-12 | -2.966e+01 | 26.08% | 13.67% | 52.2bp (69.5bp) | RO3G\_00049(RRM)/Rhizopus\_oryzae-RNCMPT00205-PBM/HughesRNA(0.927) More Information \| Similar Motifs Found | motif file (matrix) |
| 30 | T G A C T C G A T A C G C A G T A C T G T G C A T A G C T C A G C A G T G T C A C G T A A T G C A C G T | 1e-12 | -2.944e+01 | 4.51% | 0.57% | 51.0bp (65.2bp) | CRE(bZIP)/Promoter/Homer(0.778) More Information \| Similar Motifs Found | motif file (matrix) |
| 31 | G A T C A C G T A C T G A C G T C T A G A C T G A T G C | 1e-12 | -2.878e+01 | 39.41% | 24.77% | 56.8bp (62.9bp) | MET28/MA0332.1/Jaspar(0.939) More Information \| Similar Motifs Found | motif file (matrix) |
| 32 | A T G C A T G C C G T A A G T C C G T A C T A G C T A G A C T G A G T C G A T C A G T C A C G T T G A C G T A C | 1e-12 | -2.870e+01 | 2.35% | 0.09% | 43.0bp (51.1bp) | MET28/MA0332.1/Jaspar(0.667) More Information \| Similar Motifs Found | motif file (matrix) |
| 33 | T A C G C A T G A C T G A C T G C T A G G T C A A C T G A C T G G T C A C G T A A G T C C G T A | 1e-12 | -2.851e+01 | 2.55% | 0.12% | 43.1bp (51.7bp) | NCU02404(RRM)/Neurospora\_crassa-RNCMPT00238-PBM/HughesRNA(0.694) More Information \| Similar Motifs Found | motif file (matrix) |
| 34 | G C T A C G T A T C G A T A C G A T C G T C G A T C G A A T C G C G A T C A T G C T A G | 1e-12 | -2.784e+01 | 13.14% | 4.92% | 42.1bp (66.1bp) | ETS:RUNX(ETS,Runt)/Jurkat-RUNX1-ChIP-Seq(GSE17954)/Homer(0.821) More Information \| Similar Motifs Found | motif file (matrix) |
| 35 | A G T C C G T A A G T C T A G C A C G T G T A C G T C A A T C G C G T A A T C G C A T G | 1e-12 | -2.779e+01 | 3.53% | 0.34% | 59.4bp (54.7bp) | TCFL2(HMG)/K562-TCF7L2-ChIP-Seq(GSE29196)/Homer(0.693) More Information \| Similar Motifs Found | motif file (matrix) |
